# The DeSign Erasmus+ ‐project:Raising Dementia Awareness in Deaf population and Results from a Cognitive Screening Test adapted for the Austrian and Greek Deaf older adults

**DOI:** 10.1002/alz70858_106336

**Published:** 2025-12-26

**Authors:** Marianna Tsatali, Athanasia‐Lida Dimou, Tarsitsa Ntova, Antigoni Angelidou, Enrico Dolza, Doris Hoffmann‐Lamplmair, Marie‐Luise Burgstaller, Kiriaki Chatziavannidou, Ege Karar, Theodoros Goulas, Nicola Della Maggiora, Romeo Seifert, Thomas Ströbele, Bencie Woll, Joanna Atkinson, Magda Tsolaki, Birgit Teichmann

**Affiliations:** ^1^ Greek Alzheimer Association and Related Disorders, Thessaloniki, Greece, Greece; ^2^ ATHENA – Research & Innovation Information Technologies, Athens, Greece, Athens, Greece; ^3^ Association of Deaf of Northern Greece, Thessaloniki, Greece; ^4^ Network Aging Research, Heidelberg University, Heidelberg, Germany; ^5^ Istituto dei Sordi di Torino, Turin, Italy, Turin, Italy; ^6^ Krankenhaus Barmherzige Brüder, Vienna, Austria; ^7^ Krankenhaus Barmherzige Brüder, Vienna, Austria, Vienna, Austria; ^8^ ATHENA – Research & Innovation Information Technologies, Athens, Greece; ^9^ Istituto dei Sordi di Torino, Turin, Italy; ^10^ University College London, London, London, United Kingdom; ^11^ AHEPA University Hospital, Thessaloniki, Greece; ^12^ Aristotle University of Thessaloniki, Thessaloniki, Greece; ^13^ Greek Association of Alzheimer Disease and Related Disorders, Thessaloniki, Greece; ^14^ Network Aging Research, Heidelberg University, Heidelberg, Germany, Heidelberg, Germany

## Abstract

**Background:**

The paucity of data on the knowledge and attitudes of Deaf people towards dementia is well documented, as is the limited access to dementia services in this population due to lack of interpreters and absence of health professionals who use sign language (SL). Therefore, delivering dementia awareness in Deaf people is assumed as a high priority which is also related to the seeking of relevant diagnostic services. Additionally, till now there are almost no screening tools for detecting early dementia signs which are adapted in national SL.

**Method:**

To address this gap, the current Erasmus+ project aims to identify attitudes and knowledge about dementia among the Deaf population using relevant psychometric tools and subsequently create dementia raising awareness seminars, tailored to the cultural background and norms of the Deaf communities in Austria, Germany, Greece, and Italy. Secondly, the only existing Cognitive Screening Test (CST), originally adapted in British SL from Atkinson et al. (2015) for detecting dementia in Deaf older adults will be also adapted in Austrian and Greek SL.

**Result:**

Initially, results from the tools measuring dementia attitudes and knowledge will be presented. Afterwards, the design of the structured seminars delivered in Deaf communities will be also displayed. Finally, the adapted versions of the Austrian and Greek CST administered in Deaf older adults in both countries will be available.

**Conclusion:**

The final goal is to increase dementia raising awareness seminars in Deaf people from Austria, Germany, Greece and Italy, as well as achieve dementia detection in this population by providing them with diagnostic and care pathways.

